# How leader–member exchange ambivalence influences employee feedback seeking and avoidance

**DOI:** 10.1038/s41598-026-35498-2

**Published:** 2026-03-09

**Authors:** Huichi Qian, Jin Cheng

**Affiliations:** School of Management, Xiamen University, Xiamen, China

**Keywords:** LMX ambivalence, Feedback seeking, Feedback avoidance, Cognitive reappraisal, Emotional exhaustion, Psychology, Human behaviour

## Abstract

Leader–member exchange ambivalence represents a critical yet underexplored factor shaping leader–employee interaction motivation. Drawing on dual-system decision theory, this study investigates how LMX ambivalence shapes employees’ feedback strategies via both intuitive and analytical processing pathways. Using hierarchical regression and Bootstrap analyses on survey data from 306 employees in China, the findings reveal that LMX ambivalence significantly increases feedback avoidance and reduces feedback-seeking behavior. Cognitive reappraisal partially mediates the relationship between LMX ambivalence and both feedback seeking and feedback avoidance, whereas emotional exhaustion partially mediates the relationship between LMX ambivalence and feedback avoidance. Moreover, the perceived organizational political climate amplifies the positive association between LMX ambivalence and emotional exhaustion, while further intensifying the negative impact of LMX ambivalence on cognitive reappraisal. These results elucidate a systematic explanatory mechanism through which LMX ambivalence undermines employees’ motivation to engage in feedback behaviors. While offering actionable insights, these findings should be interpreted in light of the specific professional and cultural context of the sample. The study provides a solid theoretical foundation for advancing research in this domain.

## Introduction

In the VUCA era, the increased unpredictability of leadership intensifies the complexity of supervisor-subordinate relationships. Within these evolving organizational environments, ambivalence has become a common employee psychological state. Leader-Member Exchange (LMX) relationships, in particular, are a significant source of this phenomenon^1^. Drawing on Lee et al. (2019)^2^, we define LMX ambivalence as an employee’s simultaneous holding of positive and negative evaluations toward their leader. This evaluative inconsistency arises from inherent conflicts within the LMX dynamic.

First, LMX involves high interdependence. Employees rely on leaders for critical resources like support and career advancement, while leaders depend on employees for high-quality task performance and loyalty. This mutual dependence, where both parties have expectations and vulnerabilities, creates fertile ground for conflicting evaluations. For instance, a supportive leader who also imposes heavy workloads can trigger both favorable and unfavorable employee assessments, fostering ambivalence.

Second, the asymmetric power dynamics inherent in LMX exacerbate this conflict. Leaders possess evaluative authority and control over resources, enabling them to both reward and punish. This creates a tension between an employee’s desire for supportive closeness and the need for defensive professional distance. A leader acting as both a caring mentor and a strict evaluator can trigger simultaneous positive and negative reactions. This dynamic forces employees to balance pursuing autonomy with accepting control, making LMX ambivalence a profound cognitive and affective challenge.

Understanding employee behavioral responses to LMX ambivalence is crucial. While prior research has linked it to outcomes like impaired performance, there remains a theoretical gap concerning the mechanisms driving bottom-up feedback behaviors^3^. Specifically, how does this complex relational perception translate into actions like feedback seeking or avoidance? We address this by employing dual-system theory, which posits that judgment is shaped by two interacting systems: an intuitive, affective, and fast System 1, and a deliberate, cognitive, and slower System 2. The effective operation of System 2 depends on available cognitive resources, whereas System 1 operates with minimal resource demand. We propose that LMX ambivalence, as a persistent socio-emotional challenge, consumes significant cognitive resources, thereby differentially affecting the activation of these two systems and creating distinct pathways to feedback responses.

In the feedback process, feedback seeking and avoidance represent different motivational orientations. Feedback seeking is a proactive, growth-oriented behavior typically requiring the deliberative processes of System 2. In contrast, feedback avoidance is a defensive, risk-averse posture often driven by the intuitive, protective reactions of System 1. This study contributes by elucidating how LMX ambivalence, by depleting cognitive resources, inhibits System 2 functioning—manifested as reduced cognitive reappraisal—and promotes System 1 dominance, leading to emotional exhaustion. These parallel yet distinct mechanisms—emotional exhaustion reflecting System 1 and cognitive reappraisal reflecting System 2—in turn shape feedback behaviors. The dual-system framework is particularly valuable as it integrates affective and cognitive processes, providing a precise theoretical basis for examining their independent mediating effects on feedback seeking and avoidance.

Furthermore, responding to calls for context-sensitive research, we introduce organizational political climate (OPC) as a boundary condition. OPC reflects employees’ perceptions of self-serving behavior and resource contestation within the organization. As a salient threat cue, OPC can exacerbate the cognitive and socio-emotional demands of LMX ambivalence, intensifying resource depletion and moderating the affective and cognitive mechanisms through which LMX ambivalence exerts its influence^4^.

**Fig. 1:**
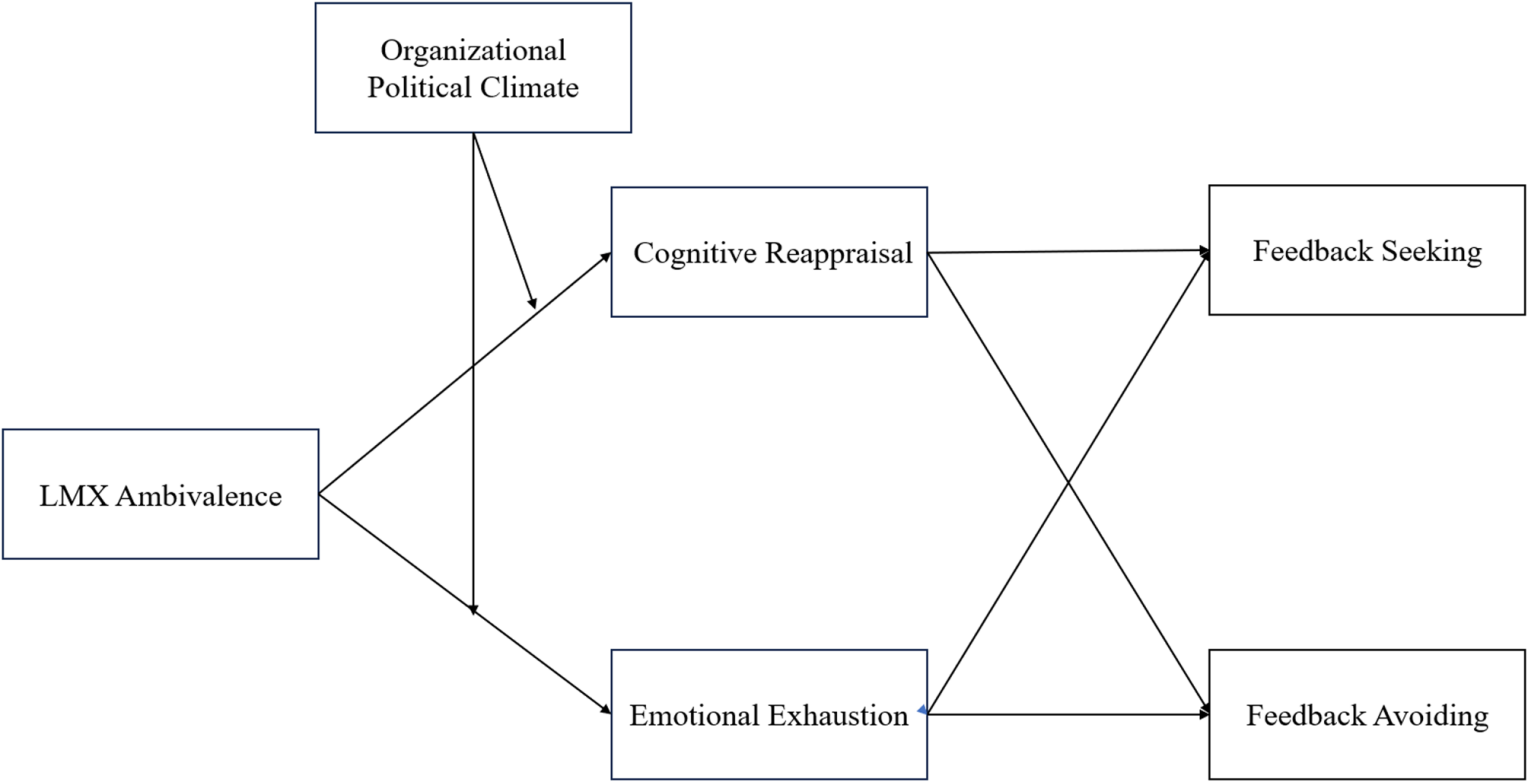
Theoretical model.

In summary, this study, grounded in dual-system theory, investigates how LMX ambivalence influences employee feedback decisions through distinct affective and cognitive pathways, with organizational political climate acting as a key boundary condition. Our research offers three main theoretical contributions. First, we advance the understanding of upward communication by integrating feedback seeking and avoidance into a unified dual-system framework. We demonstrate how LMX ambivalence, through its impact on cognitive resources, simultaneously inhibits System 2—leading to insufficient cognitive reappraisal and reduced feedback seeking—and promotes System 1, resulting in emotional exhaustion and increased feedback avoidance. Second, we refine the application of dual-system theory by parsing the interaction of affect and cognition under LMX ambivalence. By delineating specific affective (emotional exhaustion) and cognitive (cognitive reappraisal) mediators, we provide a granular explanation for how the complexity of this relational state leads to divergent behavioral decisions, directly linking resource availability to system activation. Third, by examining the moderating role of organizational political climate, we offer new insights into how contextual cues amplify the challenges of LMX ambivalence^5^, thereby shaping employees’ internal psychological processes and subsequent behavioral choices. The proposed theoretical model is depicted in Fig. 1.

## Theoretical foundation and research hypotheses

### Dual-system theory

The dual-system model of thinking proposed by Nobel laureate Daniel Kahneman has become a fundamental framework for understanding human decision-making behavior. This theory posits the existence of two interrelated systems: the intuitive system (System 1) and the analytical system (System 2). System 1 operates rapidly, automatically, and unconsciously, relying on emotional impulses, heuristics, and prior experience. It is particularly suited for handling routine or time-pressured situations but is also prone to biases and emotional distortions. In contrast, System 2 operates slowly, requires effort, and is deliberate. It involves logical reasoning and controlled information processing, enabling more reflective and rational decision-making^6^. Importantly, these two systems do not represent a strict dichotomy or mutual exclusion; rather, they typically work in concert. System 1 may generate initial judgments that are subsequently monitored and adjusted by System 2^7^. However, under conditions such as fatigue, stress, or emotional fluctuations, System 2’s capacity may become impaired, leading individuals to rely more heavily on intuitive judgments and thereby increasing susceptibility to biases and errors. This theoretical perspective provides important insights for understanding employee feedback behavior. Crucially, these cognitive and affective systems not only shape internal judgments but also serve as proximal drivers of overt behavioral responses. System 1’s fast, affect-driven judgments, such as perceiving a situation as inherently conflicting and uncomfortable, directly prompt automatic, defensive avoidance behaviors. Conversely, System 2’s deliberate, logical judgments, such as reappraising a situation as an opportunity for understanding and development, when functioning properly, can stimulate controlled, goal-oriented seeking behaviors.

### Leader-Member exchange ambivalence

LMX ambivalence arises when subordinates simultaneously hold favorable and unfavorable evaluations of their leader 9. Building on our introduction, the origins of this ambivalence stem primarily from two inherent tensions within the dyad. First, tensions from mutual interdependence: Leaders depend on employees for performance, initiative, loyalty, and vital upward communication, while employees depend on leaders for resources, support, information, and career advancement. This mutual reliance creates conflict when, for instance, a leader must be both a supportive mentor (leading to positive evaluations) and a demanding taskmaster to secure the performance they depend on (potentially leading to negative evaluations)^8^.

Second, tensions from asymmetric power dynamics: Leaders’ dual pursuit of authority and interpersonal closeness conflicts with subordinates’ simultaneous desire for support and autonomy. This dynamic creates a fundamental tension, where a leader might provide valuable guidance and support, yet their evaluative authority and control over resources—including the ability to reward or penalize—can simultaneously evoke feelings of vulnerability or constraint^9^. Power asymmetries further intensify ambivalent experiences^11^, especially when the mutual dependencies in the LMX dyad are high yet perceived as imbalanced, leading employees to navigate a complex interplay between affiliation and caution. Prior research links LMX ambivalence to diminished proactive motivation and counterproductive behaviors such as knowledge hiding^10^. Power asymmetries further intensify ambivalent experiences^11^, particularly among employees more dependent on their leaders. Recent evidence shows that LMX ambivalence frustrates core psychological needs, reduces well-being, and harms task performance, while prompting dual-norm reciprocity—where employees simultaneously engage in leader-directed helping behaviors and deviant actions.

### LMX ambivalence and feedback decision

LMX ambivalence refers to a psychological state where subordinates hold simultaneous positive and negative evaluations toward their leader. This ambivalence stems from inherent conflicting situations within LMX relationships, such as the tension between interdependence and power asymmetry. Leaders may exhibit both supportive and critical behaviors, or vacillate between delegation and micromanagement, creating evaluative inconsistencies that hinder employees from forming a stable perception of relationship quality. This persistent ambiguity constitutes a cognitive and socio-emotional challenge^12^, requiring sustained mental effort to interpret and respond, thereby placing demands on employees’ cognitive capacity.

We centrally draw upon dual-system theory, which posits that human decision-making is driven by two distinct but interacting systems. System 1 is characterized by fast, automatic, intuitive, and affect-laden processing, operating with minimal demand on cognitive resources. System 2 is slower, more deliberate, controlled, and logic-based, involving conscious reasoning and effortful cognitive operations, whose effective functioning is highly dependent on cognitive capacity. LMX ambivalence, as a complex and challenging social signal, differentially affects the activation and efficacy of these two systems primarily through its impact on the availability of employees’ cognitive resources.

We argue that the persistent and conflicting nature of LMX ambivalence chronically depletes employees’ cognitive resources. Sustained vigilance, interpretation of mixed signals, and coping with relational inconsistencies are mentally taxing, leading to a state of insufficient cognitive resources. Within the dual-system framework, this depletion profoundly impacts decision-making. Specifically, when cognitive resources are challenged or insufficient, the effortful and resource-intensive System 2 is inhibited. Consequently, the individual’s ability to engage in complex, deliberate thought processes declines. In this state, the cognitively more economical and automatic System 1 tends to dominate behavioral responses. System 1, inherently prioritizing immediate and less effortful solutions, naturally inclines towards behaviors that minimize cognitive load. Feedback avoidance, as a low cognitive effort mechanism^13^, aligns perfectly with this System 1 dominance. By choosing to avoid potentially stressful or ambiguous feedback interactions, employees can minimize further cognitive and emotional expenditure, thereby conserving their depleted resources and disengaging from situations requiring more effortful processing. This stems directly from System 1’s preference for low-effort, immediate responses when cognitive resources are scarce.

Feedback seeking is inherently a proactive and resource-intensive behavior requiring deliberate planning, strategic question formulation, and the courage to initiate potentially uncomfortable interactions—functions primarily associated with System 2^14^. When LMX ambivalence depletes cognitive resources, System 2’s capacity to engage in such effortful processes is impaired. Employees find it more difficult to consciously reinterpret ambiguous situations (i.e., cognitive reappraisal) or muster the mental energy needed to initiate proactive information gathering. Although LMX ambivalence itself creates a need for clarity (a System 2 goal), when System 2 is inhibited, the mental cost and cognitive effort required to overcome System 1’s defensive tendencies and initiate seeking from an ambivalent leader become prohibitively high. The ongoing mental exertion of managing the ambivalent relationship, coupled with the inherent psychological risk of seeking feedback from a leader sending mixed signals, ultimately makes proactive feedback seeking less likely. Thus, even if an initial desire for clarity exists, the overall reduced capacity of System 2 to execute complex, proactive strategies leads to decreased feedback-seeking behavior.

Based on this, the following hypotheses are proposed for this research:

H1: LMX ambivalence positively predicts employee feedback avoidance.

H2: LMX ambivalence negatively predicts employee feedback-seeking.

### The mediating role of emotional exhaustion

Emotional exhaustion, characterized by feelings of being emotionally overextended and depleted, arises from prolonged exposure to interpersonal stressors. Leader-subordinate relationships’ recurring and asymmetric nature makes LMX ambivalence—defined as employees’ simultaneous positive and negative evaluations of their leader—a persistent psychological strain^15^. This ambivalence demands continuous effort to monitor and interpret contradictory cues, a burden predominantly processed by System 1 that culminates in emotional exhaustion^16^. Such exhaustion represents the cumulative negative emotional experience from System 1’s sustained vigilance in processing LMX-induced internal conflict, depleting psychological resources and manifesting as pervasive weariness. Although often linked to task-related stressors, relational ambiguity with authority figures proves equally draining. LMX ambivalence’s unpredictability necessitates constant vigilance to reconcile conflicting information, gradually accumulating negative emotions and weakening adaptive coping. This System 1-driven processing ultimately leads to emotional exhaustion.

Drawing on dual-system theory^17^, we posit emotional exhaustion as a critical mediator. As System 1 remains activated processing LMX ambivalence, its cumulative effect creates emotional exhaustion that amplifies System 1’s automatic inclination to avoid further conflict^18^. Employees thus adopt behaviors offering immediate relief, with feedback avoidance exemplifying this System 1-driven response to minimize additional emotional burden.

Furthermore, this emotional buildup impacts feedback seeking. The mental weariness from chronic System 1 processing reduces motivation and capacity for demanding behaviors like seeking developmental feedback, which requires evaluation openness and ambiguity resilience—capacities compromised when System 1 dominates^16^. Thus, emotional exhaustion also curtails System 2-driven feedback-seeking behaviors.

Based on this reasoning, we propose the following hypotheses:

H3: Emotional exhaustion mediates the relationship between LMX ambivalence and feedback avoidance.

H4: Emotional exhaustion mediates the relationship between LMX ambivalence and feedback seeking.

### The mediating role of cognitive reappraisal

Dual-system theory posits that individuals process information through intuitive (System 1) and deliberative (System 2) systems. Under relational uncertainty, such as LMX ambivalence, the intuitive system tends to dominate. Conversely, activating the deliberative system—including cognitive reappraisal—demands greater cognitive capacity. Cognitive reappraisal, an emotion regulation strategy involving the reinterpretation of affective events^19^, is a core System 2 function requiring deliberative thought, logical reasoning, and active mental construction. Because it demands attentional control and working memory, reappraisal is more likely when System 2’s cognitive resources are sufficient and not depleted by competing demands.

LMX ambivalence, marked by coexisting positive and negative signals in the supervisor-subordinate relationship, often leads to inconsistent expectations and ambiguous evaluations^20^. Such ambiguity necessitates significant cognitive effort as employees constantly attempt to reconcile contradictory cues and internal conflicts^21^. This sustained cognitive effort directly consumes and diminishes System 2’s processing capacity while fostering a heightened sense of interpersonal insecurity. This state of internal conflict impedes System 2’s ability to execute higher-order regulatory strategies like cognitive reappraisal^22^, which require mental clarity and focused deliberation. Thus, LMX ambivalence impairs System 2’s functional capacity, reducing the likelihood of deploying effortful cognitive regulation.

As a System 2 function, cognitive reappraisal enables employees to reinterpret stressful workplace experiences more constructively, potentially fostering approach-oriented behaviors like feedback seeking. When individuals effectively regulate negative emotions through System 2 processing, they are more inclined to view feedback as a developmental opportunity rather than a threat. Conversely, when LMX ambivalence constrains System 2’s reappraisal capacity, feedback is more likely to be perceived as evaluative and risky, as unmodulated System 1 responses take precedence, resulting in avoidance or inhibition of seeking. Prior research supports this link between reappraisal and proactive behavior under stress^23^.

Therefore, we propose that cognitive reappraisal mediates the relationship between LMX ambivalence and feedback behaviors. When the cognitive demands of LMX ambivalence inhibit System 2’s capacity for reappraisal, employees are less able to reframe the act of seeking feedback—inherently risky due to potential ambiguous or negative information—as a constructive opportunity. Instead, they become more susceptible to System 1’s initial discomfort or threat perceptions. This reduction in System 2’s reappraisal ability consequently hinders proactive engagement in feedback-seeking behaviors.

Based on this analysis, we propose the following hypotheses:

H5: Cognitive reappraisal mediates the relationship between LMX ambivalence and feedback avoidance.

H6: Cognitive reappraisal mediates the relationship between LMX ambivalence and feedback seeking.

### The moderating role of organizational politics

Organizational political climate (OPC) reflects shared employee perceptions of self-serving behavior, manipulation, and unofficial influence tactics in the workplace^24,25^. This salient contextual feature amplifies relational ambiguity, threatens psychological security, and undermines trust in social exchanges, fundamentally shaping how employees interpret leader-member ambivalence.

LMX ambivalence creates inherent uncertainty about leader intentions and relationship trajectories^26^, increasing reliance on contextual cues. In politically charged environments, OPC heightens sensitivity to potential manipulation, making ambiguous supervisory behaviors appear more suspicious and threatening. Through dual-system theory, we demonstrate OPC moderates LMX ambivalence’s impact on feedback behaviors by altering System 1-System 2 dynamics.

High OPC constitutes an additional pervasive source of internal conflict^24^, introducing more ambiguity and perceived threat. This instability reinforces System 1’s vigilance against potential threats. When combined with LMX ambivalence, heightened System 1 activity exacerbates negative emotional accumulation and cognitive burden, intensifying emotional exhaustion while strengthening automatic feedback avoidance tendencies. Seeking feedback from ambivalent leaders in such environments represents heightened risk^24^, triggering System 1’s conflict avoidance mechanisms.

Simultaneously, high OPC significantly impairs System 2’s capacity for deliberative processing and cognitive reappraisal. The sustained cognitive load required to navigate political environments—deciphering hidden agendas and self-protection—further depletes System 2 capacity beyond LMX ambivalence’s effects. This dual impairment makes constructive reinterpretation of ambiguous interactions particularly difficult, reinforcing negative perceptions while undermining System 2-dependent adaptive strategies^27^, including proactive feedback seeking.

Thus, high OPC intensifies LMX ambivalence’s effects by both exacerbating System 1’s emotional processing and avoidance tendencies, and further inhibiting System 2’s cognitive reappraisal capacity and proactive engagement—creating a dual-pathway amplification through reinforced System 1 activation and additional consumption of System 2’s limited cognitive resources.

Based on this analysis, we propose the following hypotheses:

H7: Organizational political climate negatively moderates the relationship between LMX ambivalence and cognitive reappraisal, such that the negative effect of LMX ambivalence on cognitive reappraisal is stronger when the political climate is high.

H8: Organizational political climate positively moderates the relationship between LMX ambivalence and emotional exhaustion, such that the positive effect of LMX ambivalence on emotional exhaustion is stronger when the political climate is high.

## Methods

### Participants and procedure

To ensure the representativeness and scientific rigor of the data, this study utilized a mixed-methods approach for data collection (both online and offline). The offline research team collaborated with management teams from multiple companies and employed snowball sampling, leveraging the research team’s social networks (e.g., MBA student groups and professional circles) to recruit participants. The online survey was distributed and collected through a professional platform, with automatic filtering to exclude responses with abnormally fast submission times, ensuring data quality. After providing an explanation of the survey’s background and assuring confidentiality, a total of 378 questionnaires were distributed. Following attention checks for incomplete responses and extreme outliers, 72 invalid responses were excluded, resulting in a final sample of 306 valid questionnaires. The participants’ ages ranged from 18 to 55 years.

To address concerns regarding potential systematic bias, a dropout analysis was conducted. The results indicated no significant differences in baseline characteristics between the 72 excluded participants and the 306 retained respondents, suggesting that the exclusion did not introduce systematic bias.

### Measurements

This research used mature scales to measure various variables and used the “translation back translation” method to determine the Chinese scale. All variables were scored using the Likert 7-point scale (1 = strongly disagree, 7 = strongly agree).

Leader member exchange (LMX) ambivalence: Using the 7-item scale developed by Lee A et al. (2019)^2^. For example, “I have conflicting thoughts: some times I think that my working relationship with my manager is very good, while at other times I don’t”, Cronbach’s α = 0.925.

Emotional exhaustion: Using the the 9-item scale developed by Maslach et al. (1981)^28^, sample items include “I feel burned out from my work.” Cronbach’s α = 0.895.

Feedback avoidance was measured using the the 6-item scale developed by Moss et al. (2003)^29^, with sample items such as “After performing poorly, I would try to schedule outside appointments to avoid my supervisor.” Cronbach’s α = 0.856.

Feedback seeking was measured using the the 5-item scale developed by Moss et al. (2003)^29^, with sample items such as “After performing well, I would ask my supervisor about my performance to draw his/her attention to my success.” Cronbach’s α = 0.930.

Organizational political climate was measured using the the 6-item scale developed by Hochwarter et al. (2003)^25^, with sample items such as “There is a lot of self-serving behavior going on.” Cronbach’s α = 0.884.

Cognitive reappraisal was measured using the the 10-item scale by Gross and John (2003)^30^, with sample items such as “When I’m faced with a strong situation, I make myself think about it in a way that helps me stay calm.” Cronbach’s α = 0.795.

Control variables: This research selected the age, gender, education level, years of work, nature of the organization, and job type of the members as control variables. Based on previous research, gender, age, education level, length of service, and job level can affect individual motivation Differences in traits and attitudes can affect employee feedback behavior^31,32^. Therefore, this research will use these factors as control variables.

## Research results

### Validity testing and common method bias testing

This research conducted confirmatory factor analysis using Amos 22.0 to test the discriminant validity of six factors: LMX Ambivalence, cognitive reappraisal, emotional exhaustion, feedback avoidance, feedback seeking, and organizational political climate. This study employed confirmatory factor analysis (CFA) to assess the fit of several measurement models. The six-factor model exhibited the best fit, with all indices surpassing recommended thresholds. Specifically, it yielded a CMIN/DF of 1.809, well below the 3.0 threshold, indicating strong model parsimony. The Tucker–Lewis Index (TLI) and Comparative Fit Index (CFI) were 0.912 and 0.919, both above the ideal 0.90 benchmark, while the root mean square error of approximation (RMSEA) was 0.052, well within the acceptable range (below 0.08). In contrast, the five-factor model (with emotional exhaustion and cognitive reappraisal combined into one factor) yielded a CMIN/DF of 2.851, with TLI = 0.799, CFI = 0.812, and RMSEA = 0.078—still acceptable, but inferior to the six-factor model. Overall, the six-factor model is the most parsimonious and theoretically sound representation of the observed variables, supported by the CFA results.

Regarding the common method bias, on the one hand, anonymous questionnaires were used for questionnaire collection, while reverse questions and other methods were added for pre control; On the other hand, we conducted Harman’s single-factor test. The results showed that the first unrotated factor accounted for 28.608% of the total variance, which is below the recommended threshold of 40%, indicating that common method bias is unlikely to be a major concern. Furthermore, we employed the Latent Methods Factor (LMF) approach using AMOS^33^. The model including the latent method factor yielded a slightly improved fit (CFI = 0.924) compared to the baseline model without the method factor (CFI = 0.919). This minimal change in model fit indicates that the influence of common method variance is likely negligible^33,34^. Together, these results suggest that common method bias is not a significant threat to the validity of our findings.

### Relevant analysis and testing

Table 1 demonstrates the means, standard deviations, and correlation coefficients of each variable in Study. Among them, there was a significant positive correlation between the LMX ambivalence and Feedback seeking (*r*=-0.198, *P* < 0.001), and a significant negative correlation with Feedback avoidance (*r* = 0.455, *P* < 0.001). The above results are consistent with theoretical expectations and provide preliminary support for subsequent hypotheses.

**Table 1:** Correlation Analysis.

	1	2	3	4	5	6	7	8	9	10	11	12
Gender	1											
Age	0.032	1										
Education Level	0.075	-0.037	1									
Working Seniority	-0.057	0.648*	0.045	1								
Unit Nature	0.104	0.065	0.166	-0.085	1							
Job Type	0.001	0.356*	-0.028	0.371*	-0.082	1						
LMX Ambivalence	-0.095	-0.130*	-0.125*	-0.067	-0.003	0.014	1					
Cognitive Reappraisal	-0.029	0.112	0.068	0.08	-0.002	-0.004	-0.426*	1				
Emotional Exhaustion	-0.126*	-0.084	-0.087	-0.032	-0.045	-0.058	0.510*	-0.404*	1			
Feedback Seeking	0.01	-0.009	0.033	-0.076	0.078	-0.047	-0.198*	0.224*	-0.185	1		
Feedback Avoidance	-0.07	-0.181	-0.09	-0.142*	-0.01	-0.079	0.455*	-0.442*	0.552*	-0.181	1	
Organizational Political Climate	-0.052	-0.107	-0.103	0.026	-0.083	-0.041	0.297*	-0.279*	0.319*	-0.091	0.323*	1
M	1.549	3.144	2.059	3.333	2.209	1.987	2.461	5.850	2.066	5.037	2.090	2.702
SD	0.498	1.428	0.540	0.810	1.255	1.031	1.134	0.672	0.761	1.355	0.787	0.960

Note: * represents *P* < 0.05, * * represents *P* < 0.01, and * * * represents *P* < 0.001 (double tailed).

### Hypothesis testing

#### Testing of main and mediating effects

This study employed hierarchical regression and the Bootstrap method to examine the mediating effect of cognitive reappraisal, and the corresponding results are presented in Table 2. In Model 1, LMX ambivalence had a significant effect on feedback seeking (β = −0.202, *p* < 0.001), supporting Hypothesis H2 and indicating a significant negative relationship between LMX ambivalence and feedback seeking. In Model 2, after including cognitive reappraisal as a mediating variable, the direct effect of LMX ambivalence on feedback seeking was reduced, and the effect of cognitive reappraisal on feedback seeking was significant (β = 0.175, *p* < 0.01). Further Bootstrap testing indicated that the mediation effect was significant (indirect effect = − 0.088, 95% CI = [− 0.151, − 0.012], excluding zero), with the mediation effect accounting for 36.285% of the total effect, demonstrating that cognitive reappraisal mediates the relationship between LMX ambivalence and feedback seeking. Hypothesis H6 was thus supported.

In Model 3, LMX ambivalence had a significant effect on feedback avoidance (β = 0.435, *p* < 0.001), supporting Hypothesis H1 and indicating a significant positive relationship between LMX ambivalence and feedback avoidance. In Model 4, after adding cognitive reappraisal as a mediating variable, the direct effect of LMX ambivalence on feedback avoidance was reduced, and the effect of cognitive reappraisal on feedback avoidance was significant (β = −0.300, *p* < 0.001). Further Bootstrap testing confirmed that the mediation effect was significant (indirect effect = 0.088, 95% CI = [0.032, 0.226], excluding zero), with the mediation effect accounting for 28.977% of the total effect, indicating that cognitive reappraisal mediates the relationship between LMX ambivalence and feedback avoidance. Hypothesis H5 was therefore supported.

**Table 2:** Mediation test for cognitive Reappraisal.

Variable	Feedback Seeking		Feedback Avoidance	Feedback Avoidance	Cognitive Reappraisal
	Model 1	Model 2	Model 3	Model 4	Model 5
Gender	-0.023	-0.011	-0.026	-0.047	-0.071
Age	0.032	0.022	-0.081	-0.064	0.057
Education level	0.004	0.000	-0.035	-0.029	0.022
Working Seniority	-0.102	-0.105	-0.046	-0.040	0.019
Unit Nature	0.067	0.068	-0.002	-0.003	-0.004
Job Type	-0.012	-0.008	-0.041	-0.048	-0.025
LMX Ambivalence	-0.202***	-0.129*	0.435***	0.309***	-0.421***
Cognitive Reappraisal		0.175**		-0.300***	
R^2^	0.053	0.078	0.228	0.300	0.191
ΔR^2^	0.053	0.025	0.228	0.073	0.191
F	2.399***	3.143***	12.545***	15.926***	10.051***

This study employed hierarchical regression and the Bootstrap method to examine the mediating effect of cognitive reappraisal, and the relevant results are shown in Table 3. In Model 7, after including emotional exhaustion as a mediating variable, the direct effect of LMX ambivalence on feedback avoidance was reduced, and the effect of emotional exhaustion on feedback avoidance was significant (β = 0.430, *p* < 0.001). This result indicates that emotional exhaustion mediates the relationship between LMX ambivalence and feedback avoidance. Further Bootstrap testing showed that the mediation effect was significant (indirect effect = 0.150, 95% CI = [0.104, 0.337], excluding zero), with the mediation effect accounting for 49.708% of the total effect, confirming that emotional exhaustion plays a significant mediating role between LMX ambivalence and feedback avoidance. Hypothesis H3 was therefore supported.

In Model 9, after including emotional exhaustion as a mediating variable, the effect of LMX ambivalence on feedback seeking was not significant (β = −0.114). This result suggests that emotional exhaustion does not mediate the relationship between LMX ambivalence and feedback seeking. Thus, Hypothesis H4 was not supported.

**Table 3:** Mediation test for emotional Exhaustion.

Variable	Feedback Avoidance		Feedback Seeking		Emotional Exhaustion
	Model 6	Model 7	Model 8	Model 9	Model 10
Gender	-0.026	0.004	-0.023	-0.031	-0.071
Age	-0.081	-0.079	0.032	0.032	-0.004
Education Level	-0.035	-0.028	0.004	0.002	-0.017
Working Seniority	-0.046	-0.057	-0.102	-0.099	0.027
Unit Nature	-0.002	0.014	0.067	0.063	-0.037
Job Type	-0.041	-0.008	-0.012	-0.021	-0.077
LMX Ambivalence	0.435***	0.219***	-0.202***	-0.145*	0.504***
Emotional Exhaustion		0.430***		-0.114	
R^2^	0.228	0.362	0.053	0.063	0.273
ΔR^2^	0.228	0.134	0.053	0.009	0.273
F	12.545***	21.053***	2.399*	2.487*	15.978***

Note: * represents *P* < 0.05, * * represents *P* < 0.01, and * * * represents *P* < 0.001 (double tailed).

#### Testing the moderating effect of organizational political climate

In this study, LMX ambivalence was specified as the independent variable, organizational political climate as the moderating variable, and gender, age, education level, and position as control variables. All independent and moderating variables were mean-centered prior to creating interaction terms to mitigate potential multicollinearity. As shown in Table 4 (Models 12 and 13), LMX ambivalence exerted a significant positive effect on emotional exhaustion (β = 0.121, *p* < 0.01), and the interaction term between LMX ambivalence and organizational political climate was also positively associated with emotional exhaustion (β = 0.101, *p* < 0.01). This indicates that a higher level of organizational political climate strengthens the positive relationship between LMX ambivalence and emotional exhaustion, thereby supporting Hypothesis 8. Furthermore, LMX ambivalence had a significant negative effect on cognitive reappraisal (β = −0.105, *p* < 0.001). The interaction between LMX ambivalence and organizational political climate was also significant (β = −0.075, *p* < 0.05), suggesting that organizational political climate amplifies the negative impact of LMX ambivalence on cognitive reappraisal. In other words, under a high organizational political climate, the detrimental influence of LMX ambivalence on cognitive reappraisal becomes more pronounced. These results provide empirical support for Hypothesis 7.

**Table 4:** Moderation test on organizational political Climate.

	Emotional Exhaustion			Cognitive Reappraisal		
	Model 11	Model 12	Model 13	Model 14	Model 15	Model 16
Gender	-0.108	-0.108	-0.093	-0.095	-0.095	-0.107
Age	-0.002	0.013	0.009	0.027	0.014	0.017
Education level	-0.024	-0.005	-0.021	0.027	0.011	0.023
Working seniority	0.025	-0.003	-0.01	0.016	0.04	0.045
Unit Nature	-0.023	-0.017	-0.028	-0.002	-0.007	0.002
Job Type	-0.057	-0.049	-0.029	-0.016	-0.022	-0.038
LMX Ambivalence	0.338***	0.305***	0.278***	-0.249***	-0.221***	-0.201***
Organizational Political Climate		0.141**	0.121**		-0.120***	-0.105***
LMX Ambivalence X Organizational Political Climate			0.101**			-0.075*
R2	0.273	0.301	0.324	0.191	0.217	0.234
ΔR^2^	0.273	0.028	0.023	0.191	0.026	0.017
F	15.978***	15.974***	15.775***	10.051***	10.288***	10.022***

**Fig. 2:**
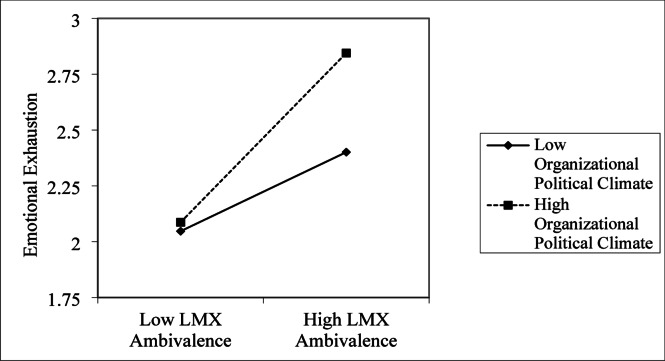
The moderating effect of organizational political climate on the LMX ambivalence and Emotional Exhaustion.

**Fig. 3:**
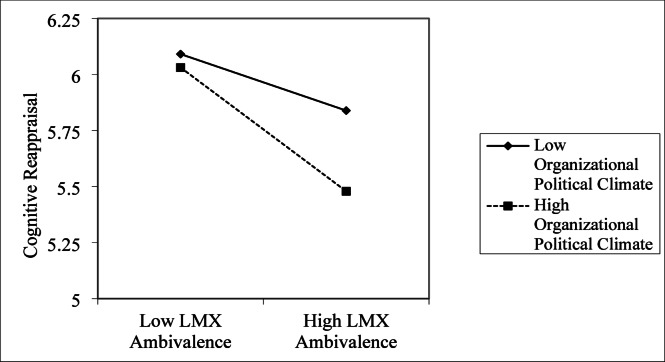
The moderating effect of Organizational Political Climate on the LMX ambivalence and Cognitive Reappraisal.

To further evaluate the moderating role of self-enhancing humor in the association between workplace isolation and problem‐solving pondering, the interaction terms were plotted and simple slope analyses were conducted. As illustrated in Fig. 2, when the Organizational Political Climate was high (M + 1SD), LMX Ambivalence showed a significant positive association with Emotional Exhaustion; under low Organizational Political Climate (M − 1SD), this relationship was not statistically significant. These findings provide empirical support for Hypothesis 8. As shown in Fig. 3, LMX Ambivalence exhibited a significant negative association with Cognitive Reappraisal. Moreover, a high Organizational Political Climate (M + 1SD) intensified this negative relationship, whereas under low Organizational Political Climate (M − 1SD), the association was weaker and not significant. This pattern of results supports Hypothesis 7.

**Fig. 4:**
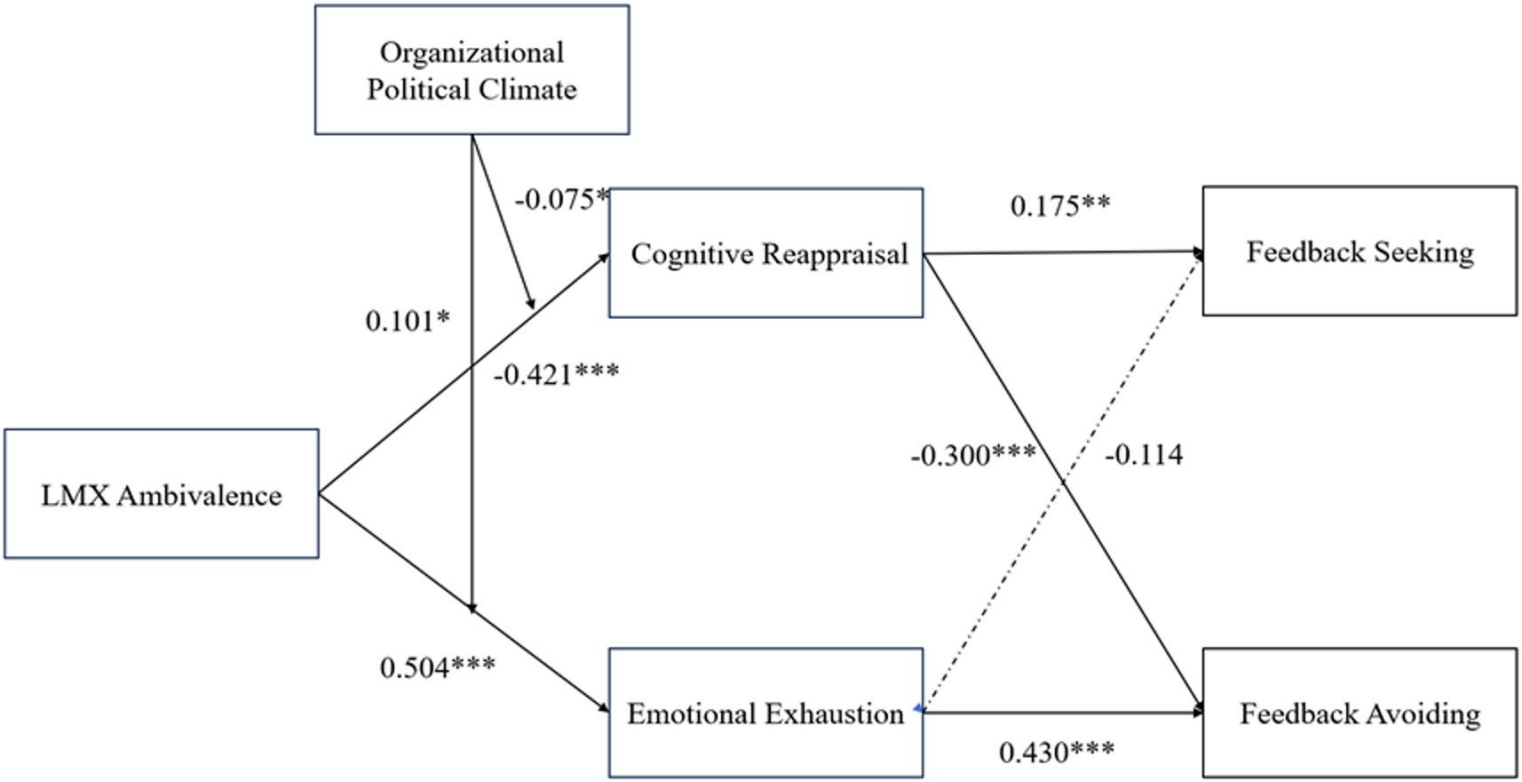
The final model of this study. Notes: *p < 0.05. *p* < 0.01. *p < 0.001.

As shown in Fig. 4, the final structural model presents all standardized path coefficients for the hypothesized relationships, including both direct and moderated effects. The diagram visually summarizes the strength and direction of each path within the research framework, providing an integrated view of the tested hypotheses and their statistical significance.

## Discussion

### Theoretical implications

First, this study integrates the dual-system theory perspective into the LMX and feedback-seeking literature, explaining how cognitive (System 2) and affective (System 1) processes jointly link LMX ambivalence with feedback behaviors, thereby extending related research. Compared to previous research that primarily examined the buffering role of social support in task performance^2^, our findings reveal that LMX ambivalence reduces cognitive reappraisal, thereby decreasing feedback seeking and increasing feedback avoidance; simultaneously, it exacerbates emotional exhaustion, which strengthens feedback avoidance but does not significantly affect feedback seeking. By going beyond simply mapping cognitive and affective pathways to different feedback outcomes in a one-to-one manner, and by integrating both feedback seeking and avoidance into this framework^35^, we provide an explanatory model that captures the dynamic complexity of ambivalent relationships, offering mechanistic insights into how conflicting supervisor-subordinate perceptions affect workplace behaviors.

Second, this study provides important theoretical insights, particularly through the unexpected non-significant mediating role of emotional exhaustion in the relationship between LMX ambivalence and feedback seeking. Although non-significant results are often viewed as research limitations, we argue that they offer valuable theoretical implications by optimizing the application of dual-system theory in organizational contexts. Our results show that emotional exhaustion mediates the relationship between LMX ambivalence and feedback avoidance, but not between LMX ambivalence and feedback seeking; whereas cognitive reappraisal mediates both feedback behaviors. This pattern suggests that the two forms of feedback behaviors may be driven by different psychological processes. Feedback seeking typically requires deliberate, effortful cognitive processing (System 2), while feedback avoidance is more automatic and self-protective (System 1). Emotional exhaustion—reflecting the depletion of affective resources—appears to be more strongly associated with automatic^36^, low-deliberation behaviors (such as feedback avoidance), while its impact on cognitively effortful behaviors (such as feedback seeking) is less evident in the data. Another possibility is that other cognitive or situational factors mitigate the effect of emotional exhaustion on feedback seeking, thus weakening its mediating role.

By identifying this differential pattern, this study helps clarify the boundary conditions under which emotional exhaustion influences behavior. Specifically, it seems more likely to shape behaviors that require minimal cognitive deliberation, while having relatively little impact on cognitively demanding behaviors. Future research could examine the interaction between emotional exhaustion and cognitive reappraisal under different organizational and individual conditions^37^, thereby advancing a more precise understanding of how LMX ambivalence translates into different feedback behaviors.

Third, this study advances contextual theory by providing a mechanistic explanation for the moderating role of organizational political climate. Rather than merely identifying political climate as a boundary condition, this study theoretically elaborates the psychological processes through which political perceptions alter employees’ threat detection systems and resource conservation strategies. Organizational political climate is conceptualized as an environmental cue that amplifies sensitivity to relational threats^38^, thereby strengthening the protective mechanisms triggered by LMX ambivalence. In a high political climate, employees become more vigilant about interpersonal risks, making them more responsive to ambivalent relational signals and more inclined to adopt defensive feedback avoidance^39^. This framework explains why political climate specifically moderates the emotional exhaustion-feedback avoidance path: politically charged environments activate threat detection systems, making emotionally exhausted employees particularly prone to withdrawal behaviors. By elucidating these mechanisms, this study positions political climate as an explanatory construct, clarifying how situational factors interact with psychological states to shape workplace behaviors^26^.

These findings contribute to the dual-system theory literature by examining the effects of relational conflict on cognitive reappraisal and emotional exhaustion. Although relational conflict always impairs cognitive reappraisal and exacerbates emotional exhaustion, the relative influence of System 1 and System 2 likely varies across individuals and contexts. Drawing on dual-system theory, we hypothesize that individual factors such as emotional intelligence^40^, self-control^41^, and psychological flexibility^42^, as well as situational factors such as reduced time pressure^43^ and higher psychological safety^44^, may activate System 2 processes, potentially mitigating the behavioral effects of emotional exhaustion. Future research should empirically test the moderating effects of these factors in similar contexts^45^. Given the Chinese cultural context of the sample, interpretations of LMX ambivalence and feedback behaviors must consider Confucian values and high power distance^46^. These cultural norms emphasize hierarchy^47^, relational obligations^48^, and harmony^49^, which may attenuate the cognitive dissonance^50^ typically associated with ambivalence and influence feedback seeking behaviors, making them more perceived as signals of loyalty rather than paths to self-improvement^51^. Feedback avoidance may aim to maintain relational harmony rather than evade criticism. These dynamics suggest that the mechanisms identified in this study might manifest differently in low power distance cultures^52,55^, where direct feedback is more normative and ambivalence may provoke confrontational responses. Cross-cultural research^53^ could further explore whether the asymmetric effects of emotional exhaustion and cognitive reappraisal are consistent across cultural contexts or depend on values emphasizing hierarchy versus harmony^54^.

### Practical implications

The findings of this study provide concrete and actionable guidance for organizational management practices, aiming to improve leader-subordinate relationships, optimize the feedback ecosystem, and enhance organizational health through multi-level intervention measures.

First, the results of this study emphasize the necessity of proactively managing LMX ambivalence through leadership development programs. As LMX ambivalence is the root cause of the problem, organizations should prioritize “reducing relational ambivalence” as a core objective of leadership training. Specific training modules should focus on enhancing managers’ awareness of consistency and role awareness, helping them recognize potential conflicts between their supportive behaviors (such as caring for subordinates) and controlling behaviors (such as performance pressure) in employees’ perceptions. Tools such as role-playing or 360-degree feedback can be used to train leaders in consistent communication techniques— for example, clearly explaining the developmental value when assigning challenging tasks, thus reframing pressure (a potential negative signal) into an opportunity (a positive support signal). Additionally, the training should strengthen leaders’ ability to perceive employees’ emotional states^56^. When signs of emotional exhaustion or avoidance are identified, managers should adopt differentiated communication strategies^57^, such as initiating informal, low-pressure conversations, rather than solely relying on formal performance evaluations, to proactively rebuild psychological safety.

Second, given that the organizational environment (particularly the political climate) significantly amplifies the negative effects of LMX ambivalence, systemic HR policy interventions are crucial. HR departments should regularly employ tools such as organizational political climate perception surveys for diagnostics, and based on the results, implement transparency reforms. For instance, publicly disclosing criteria for promotions, distribution of performance rewards, and ensuring procedural fairness in decision-making processes can effectively reduce defensive attitudes stemming from uncertainty about information, thereby buffering the threats posed by a high-political climate. Simultaneously, to directly address employees’ emotional exhaustion and cognitive resource depletion, organizations should establish a multi-dimensional support system. This should include promoting Employee Assistance Programs (EAPs) to offer professional psychological counseling services, as well as establishing informal support networks, such as Peer Coaching, which allow employees to share emotional burdens in safer, more egalitarian relationships, thereby effectively alleviating feedback avoidance tendencies.

Finally, this study reveals a clear tendency for employees to avoid feedback when experiencing ambivalence. This means organizations should not passively wait for employees to initiate communication but should design guiding, structured feedback protocols. In addition to traditional performance evaluations, managers should introduce a “Feedforward” mechanism. This dialogue model focuses on “how to do better in the future” rather than “what went wrong in the past,” which can effectively reduce employees’ defensive attitudes and shift the conversation from evaluation to development, thus encouraging feedback seeking. Furthermore, organizations must establish low-risk feedback channels. In addition to traditional open-door policies, setting up anonymous online suggestion boxes or appointing neutral HR Business Partners (HRBP) or internal coaches as feedback intermediaries can provide employees who feel threatened with a secure way to express their concerns, effectively preventing a complete breakdown of communication due to relational conflicts.

### Research limitations and future directions

While this study offers valuable insights into the relationship between leader-subordinate relationship conflict and feedback behaviors, several limitations warrant attention.

Firstly, the cross-sectional design of this study limits the ability to make definitive claims about causality and temporal sequencing. To address this, future research could utilize longitudinal models or diary study designs to examine the dynamic fluctuations of emotional exhaustion and cognitive reappraisal over time. Such an approach could reveal how these fluctuations influence feedback seeking and feedback avoidance behaviors, and explore whether LMX ambivalence accumulates over time, thus shaping these feedback behaviors in the long term.

Secondly, although emotional exhaustion and cognitive reappraisal were examined as parallel mediators in this study, future work could expand this framework by exploring additional mediators. Variables such as rumination or psychological safety may play significant roles in linking leader-subordinate relationship conflict to feedback behaviors. Furthermore, future research should explore a broader set of potential moderators—such as trust in the leader or feedback orientation—that may shape the relationship between conflict and feedback behaviors under different situational conditions.

While this study found preliminary support for the moderating role of organizational political climate on the relationship between leader-subordinate conflict and individual outcomes, further research is needed to explore the boundary conditions of this effect. Organizational culture or leadership style, for instance, may produce varying influences across different organizational contexts, potentially leading to more complex effects on individuals’ emotions and feedback behaviors.

Lastly, to enhance the generalizability of the findings and address the limitations identified, future research should prioritize diversity in terms of geography, industry, and methodology. Expanding participant samples to include regions beyond China would capture a broader range of organizational contexts. Additionally, including employees from various industries and organizations of different sizes would improve the applicability of the findings. These enhancements would bolster the robustness of the results and contribute more comprehensively to management research and practice.

## Conclusions

Drawing on findings from a sample of professionals in China, this study advances theoretical understanding of Leader–Member Exchange (LMX) dynamics by examining how subordinates’ contradictory perceptions shape feedback decision-making through dual cognitive–emotional pathways. Grounded in dual-system theory, we develop an explanatory framework that specifies psychological mechanisms potentially underlying feedback behaviors in superior–subordinate relationships, thereby extending existing accounts of LMX processes.

## Data Availability

The datasets generated and/or analysed during the current study are available from the corresponding author on reasonable request.
